# Extracorporeal photopheresis vs standard therapies for steroid‐refractory chronic graft‐vs‐host disease: Pharmacoeconomic assessment of hospital resource use in Spain

**DOI:** 10.1002/jca.21901

**Published:** 2021-05-08

**Authors:** Blanca Boluda, Antonio Solana‐Altabella, Isabel Cano, Evelyn Acuña‐Cruz, Rebeca Rodríguez‐Veiga, Octavio Ballesta‐López, Juan Eduardo Megías‐Vericat, David Martínez‐Cuadrón, Ines Gómez, Pilar Solves, Ignacio Lorenzo, Jose Luis Piñana, Jaime Sanz, Manuel Guerreiro, Juan Montoro Gómez, Alvaro Díaz‐González, Javier Marco, Albert Blanco, Miguel Á. Sanz, Pau Montesinos

**Affiliations:** ^1^ Haematology Department Hospital Universitari i Politècnic La Fe Valencia Spain; ^2^ Pharmacy Department Hospital Universitari i Politècnic La Fe Valencia Spain; ^3^ Hematology Research Group Instituto de Investigación Sanitaria La Fe Valencia Spain; ^4^ CIBERONC Instituto Carlos III Madrid Spain

**Keywords:** cost comparison, extracorporeal photopheresis, graft‐vs‐host disease, healthcare resource utilization

## Abstract

**Background:**

This study assessed pharmacoeconomic costs associated with extracorporeal photopheresis (ECP) compared with other available second‐line therapies for chronic graft‐vs‐host disease (cGvHD) in a tertiary Spanish institution.

**Methods:**

Patients (≥18 years) diagnosed with steroid‐refractory cGvHD were eligible. Data were collected retrospectively from index date until 1 year or relapse. Patients were distributed in two cohorts (ECP vs non‐ECP), matched by age (≤ or > 40), hematopoietic stem cell transplant (HLA‐identical sibling donor or other) and number of previous immunosuppressive lines (1, 2, or ≥ 3). Costs were assigned using the 2016 diagnosis‐related group (DRG) system: DRG 579 (€22 383) overnight stay due to major complication (ie, sepsis, pneumonia, parenteral nutrition, or respiratory failure), and DRG 875 (€5154) if no major complication. The primary endpoint was healthcare resource utilization per patient.

**Results:**

Forty patients (n = 20 per cohort) were included. Median age was 49, and 37.5% were female. Mean total cost per patient was €25 319 (95% CI: €17 049–€33 590) across the two cohorts, with a slightly lower mean cost per ECP‐treated patient (€23 120) compared with the non‐ECP cohort (€27 519; *P* = .597). Twenty‐seven inpatient hospitalizations occurred among ECP‐treated patients, vs 33 in the non‐ECP cohort. Day hospital and external consultations were more frequent in the ECP cohort. However, fewer inpatient admissions included DRG 579 compared with the non‐ECP cohort (44% vs 58%). Inpatient length of stay was slightly shorter in the ECP cohort (30 vs 49 days; *P* = .298).

**Conclusions:**

ECP treatment may yield economic savings in Spain through resource savings and moving costs toward outpatient care.

## 1 INTRODUCTION

Chronic graft‐vs‐host disease (cGvHD) occurs in 30% to 80% of patients who have received allogeneic hematopoietic stem cell transplantation (allo‐HSCT) for diseases including leukemia and aplastic anaemia.^1^, ^2^, ^3^ Although allo‐HSCT is seen as a curative option for many patients with hematological malignancies, cGvHD remains a significant cause of long‐term morbidity and non‐relapse mortality.^1^, ^2^, ^3^, ^4^ High‐dose corticosteroids, often in combination with calcineurin inhibitors, are currently recommended as first‐line treatment for patients with cGvHD.^5^ However, up to 50% of patients do not respond, and patients with a partial response will depend on this first‐line treatment to maintain their response and can suffer relapse.^6^ Patients receiving long‐term corticosteroid treatment are subject to numerous complications and adverse effects, including substantially elevated risk of serious infections^7^; thus, dose reduction of corticosteroids has long been an unmet need and important target in rescue treatment of cGvHD.^6^, ^8^

Extracorporeal photopheresis (ECP) is an immunomodulatory leukapheresis‐based treatment, in which the patient's leukocytes are collected, treated with 8‐methoxypsoralene and ultra‐violet A light and subsequently reinfused to the patient.^9^ ECP is an accepted, safe and effective second‐line treatment for cGvHD, reducing the need for immunosuppressive agents for symptom and disease control following allo‐HSCT.^10^, ^11^, ^12^ However, knowledge of the health economic impact of these treatments in Spain is currently limited.^13^ The primary objective of this study was to assess the use of hospital resources and pharmacoeconomic costs associated with ECP compared with other standard second‐line therapies for resistant or steroid‐dependent cGvHD in a tertiary Spanish institution. Secondary objectives from this study were to evaluate the treatment response and survival associated with second‐line therapies, and to assess the time and costs of multi‐step vs integrated technologies for ECP delivery.

## 2 METHODS

### 2.1 Patients

Eligible patients were adult (≥18 years) hematopoietic stem cell transplant recipients with a clinical and/or histological diagnosis of steroid‐refractory or ‐dependent cGvHD. Refractory disease was defined as progression of signs/symptoms of GvHD, or lack of improvement after at least 2 weeks of corticosteroid treatment (prednisone ≥1 mg/kg/day), or intolerance to first‐line treatments (other immunosuppressants). Steroid‐dependent disease was defined as minor improvement of GvHD signs/symptoms with corticosteroid treatment (prednisone ≥15 mg/day), but disease improvement was not sustained, or worsened, during dose tapering. Data were collected retrospectively from all patients within a period of more than 6 years between January 1, 2010, and May 1, 2016, with a follow‐up period for each patient of 12 months from the index date (ie, date of start of treatment line for steroid‐refractory/−dependent cGvHD).

### 2.2 Study design

This retrospective, observational, pharmacoeconomic study compared a patient cohort treated with ECP to a cohort treated with other standard second‐line treatments. Eligible patients from ”Hospital Universitari i Politècnic La Fe″ in Valencia, Spain, were distributed in two cohorts depending on treatment (ECP or other standard therapies), and were matched by the following: age (≤40 or > 40 years); type of transplant (HLA‐identical sibling donor or other); number of previous immunosuppressive treatments for cGvHD (1, 2, or ≥ 3); and criteria related to cGvHD including treatment line studied, clinical severity, organ involvement, and corticosteroid resistance vs dependence.

ECP schedules for cGvHD are variable. Generally, treatment is performed on two consecutive days every week or every 2 weeks for 8 to 12 weeks, or until a response is noticeable, and is then followed by a tapering regimen with treatment performed once every 4 weeks.^14^

### 2.3 Study procedures and evaluations

Healthcare resource utilization (HCRU) included costs associated to inpatient hospitalizations due to the management of cGvHD complications and its treatment, as well as outpatient care in external consultations and day hospital visits. Number and duration of each type of care for the two cohorts were the primary variables of this pharmacoeconomic assessment. Hospital admission costs for each cohort were calculated using the 2016 Spanish national diagnosis‐related group (DRG) assignment system, and the average length of stay for each DRG code (Supplemental S1). Secondary variables included duration and costs of the steps involved in integrated vs multi‐step ECP technologies, accounting for direct and indirect costs, including physician and technical operator salaries, bed retention, procedure kits and laboratory analysis costs, material transportation and equipment maintenance; duration and cost of steps were calculated based on previous studies and our own experience. Integrated ECP was performed using CELLEX equipment (Therakos) and multi‐step ECP with Spectra Optia cell separator (Terumo BCT) and UVA‐Pit illuminator (MedTech Solutions), following the manufacturer's instructions. Other secondary variables were: cGvHD overall response rate, duration of response, overall survival and transplant‐related mortality at the first and second year from treatment initiation.

### 2.4 Statistical analysis

Statistical significance was determined by chi‐squared tests for categorical variables and student's *t* test for continuous variables. The Clopper‐Pearson method was used to determine 95% confidence intervals.^15^ Statistical analyses were performed using Stata version 14.2. The study was not designed to be statistically powered; nominal *p* values are reported.

## 3 RESULTS

### 3.1 Patient disposition and baseline characteristics

**FIGURE 1:**
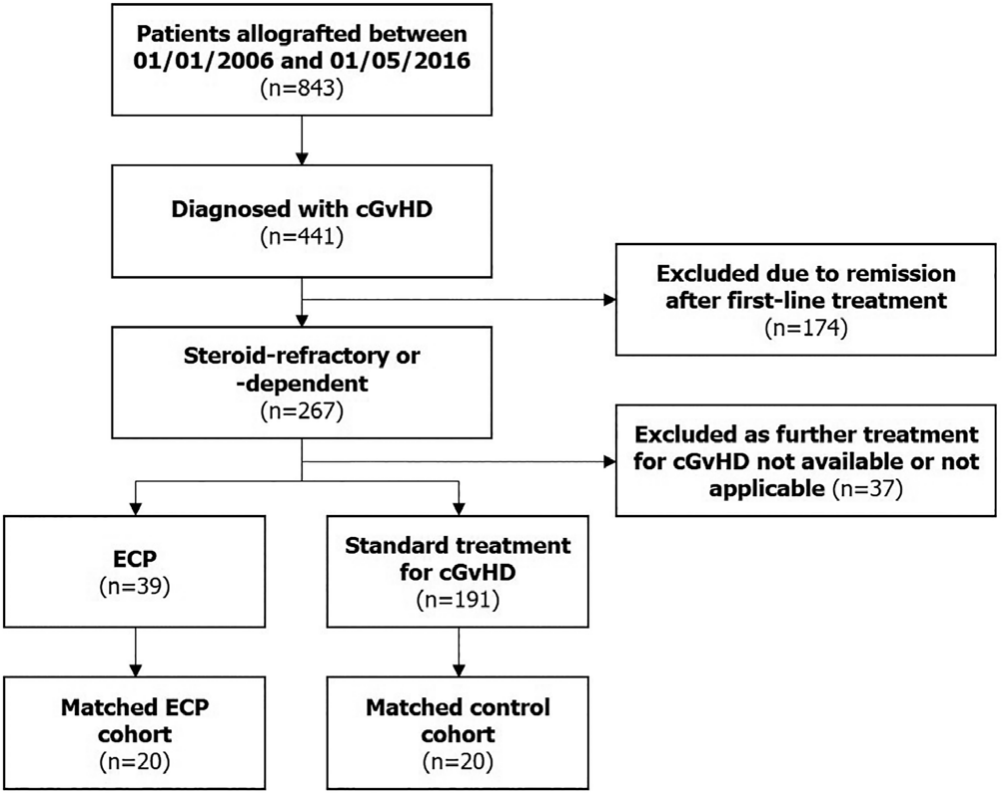
Study consort diagram. cGvHD, chronic graft‐vs‐host disease; ECP, extracorporeal photopheresis

Of the 843 patients who were screened, 40 (n = 20 per cohort) were included in this study (Figure 1). At index date, patients' median age was 49 years (20–64 years), 15 (37.5%) were female and response to first‐line steroid treatment was refractory and dependent in 18 (45%) and 22 (55%) patients, respectively. Patient baseline demographics for each cohort are summarized in Table 1; there were no significant differences in any patient characteristics between the two matched cohorts (*P* > .05). Comparing ECP and non‐ECP cohorts, 12 (60%) and 14 (70%) patients were older than 40 years, respectively; 12 (60%) and 14 (70%) patients had HLA‐identical sibling donors, respectively, and eight (40%) patients in each cohort had only one previous treatment line for cGvHD.

**TABLE 1:** Patient baseline characteristics

Characteristic	ECP (n = 20)	Non‐ECP (n = 20)	*P* value
**Female, n (%)**	8 (40)	7 (35)	.74
**Age at index date, years, median (range)**	47 (20–64)	50 (20–63)	.51
≤40 n (%)	8 (40)	6 (30)	
>40 n (%)	12 (60)	14 (70)	
**Underlying disease, n (%)**			.69
ALL	4 (20)	2 (10)	
AML/MDS	10 (50)	11 (55)	
NHL/HD/MM/CLL	4 (20)	6 (30)	
Other	2 (10)	1 (5)	
**Type of transplant, n (%)**			.51
HLA‐identical sibling	12 (60)	14 (70)	
Other	8 (40)	6 (30)	
**Type of response first line with steroids, n (%)**			.52
Refractory	8 (40)	10 (50)	
Dependent	12 (60)	10 (50)	
**Previous treatments for cGvHD, n (%)**			.92
1	8 (40)	8 (40)	
2	6 (30)	7 (35)	
≥3	6 (30)	5 (25)	
**Source of stem‐cells, n (%)**			.25
Bone marrow	1 (5)	0 (0)	
Cord blood	5 (25)	2 (10)	
Peripheral blood	14 (70)	18 (90)	
**Type of ECP, n (%)**			**‐**
Integrated	7 (35)	‐	
Two‐step procedure	13 (65)	‐	
**Concomitant treatments for cGvHD, n (%)**			.23
Steroids dose increased	4 (20)	5 (25)	
Steroids + calcineurin inhibitors	6 (30)	2 (10)	
Steroids + sirolimus +/− MMF	7 (35)	3 (15)	
Steroids + MMF	0 (0)	4 (20)	
Steroids + calcineurin inhibitors + MMF	1 (5)	1 (5)	
Steroids + thymoglobulin	0 (0)	3 (15)	
Steroids + infliximab	0 (0)	1 (5)	
Steroids + imatinib	0 (0)	1 (5)	
Ruxolitinib	2 (10)	0 (0)	

Abbreviations: ALL, acute lymphocytic leukemia; AML, acute myeloid leukemia; cGvHD, chronic graft‐vs‐host disease; CLL, chronic lymphocytic leukemia; ECP, extracorporeal photopheresis; HD, Hodgkin's disease; HLA, human leukocyte antigen; MDS, myelodysplastic syndromes; MM, multiple myeloma; MMF, mycophenolate mofetil; NHL, non‐Hodgkin lymphoma.

### 3.2 Resource utilization

**FIGURE 2:**
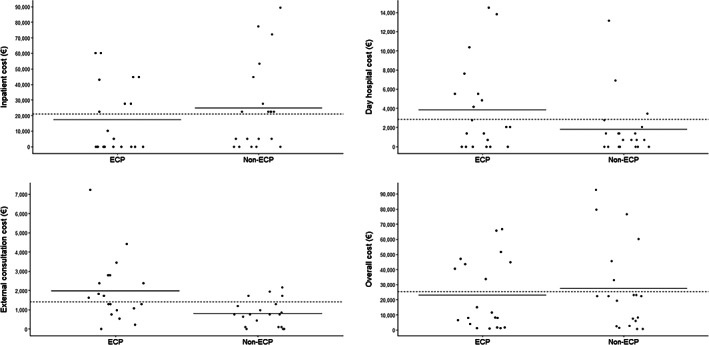
Comparison of HCRU in ECP vs non‐ECP‐treated patients. Cost breakdown for ECP vs non‐ECP‐treated patients. A, Overall cost; B, outpatient day hospital cost; C, external consultation cost; D, inpatient cost. Solid lines represent mean costs per patient of the respective cohort; dashed lines represent mean cost per patient across both cohorts. ECP, extracorporeal photopheresis; HCRU, healthcare resource utilization

In total, 60 inpatient admissions occurred during the study period totaling 1568 days spent in hospital, with a mean duration of 39.2 (95% confidence interval [CI]: 21–57) days' stay for each cohort. Mean total cost per patient was €25 319 (95% CI: €17 049–€33 590) across the two cohorts, with a slightly lower mean total cost per patient in the ECP cohort (€23 120) compared with the non‐ECP cohort (€27 519; *P* = .597; Figure 2A). In the ECP cohort, there were 27 inpatient hospitalizations (mean 1.4 per patient), while in the non‐ECP cohort, there were 33 inpatient hospitalizations (mean 1.7 per patient; *P* = .6097).

**FIGURE 3:**
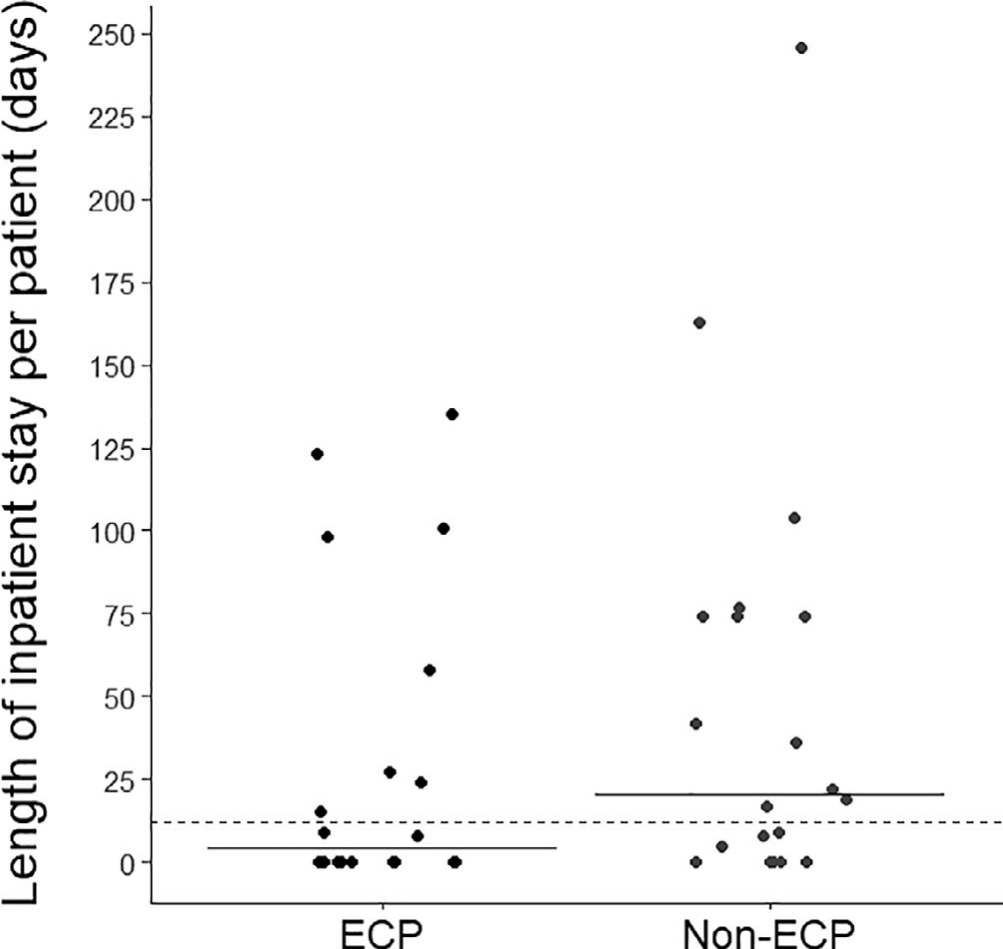
Comparison of LOS in ECP vs non‐ECP‐treated patients. Length of inpatient stay per ECP vs non‐ECP‐treated patient (solid lines represent median inpatient stay per patient of the respective cohort; the dashed line represents median inpatient stay per patient across both cohorts). ECP, extracorporeal photopheresis; LOS, length of stay

Owing to the delivery of ECP as an outpatient treatment, day hospital and external visits/consultations were more frequent in the ECP cohort (Table 2). The median (range) number of outpatient day hospital visits was 3.0 (0–19) and 1.0 (0–21) in the ECP cohort and non‐ECP cohort, respectively (*P* = .053). This difference in number of outpatient day hospital visits incurred a mean additional cost of €2005 per ECP‐treated patient (Figure 2B). Patients treated with ECP attended a median (range) of 15.0 (0–20) external consultations, compared with 7.0 (0‐67) external consultations per non‐ECP‐treated patient (*P* = .011). External consultations incurred a mean additional cost of €1171 per ECP‐treated patient (Figure 2C). However, the proportion of inpatient hospital admissions assigned DRG 579* was smaller in the ECP cohort; 44% (12/27) and 58% (19/33) of admissions included overnight stay due to major complications in the ECP cohort and non‐ECP cohort, respectively. Consequently, mean inpatient cost per patient was slightly lower in the ECP cohort compared with non‐ECP cohort (€17 295.27 vs €24 871.43; *P* = .3501; Figure 2D); median (range) total inpatient length of stay (LOS) per patient was slightly shorter in the ECP cohort (4.0 [0–246] days) compared with the non‐ECP cohort (20.5 [0–135] days; *P* = .206; Figure 3).

**TABLE 2:** Health care resource utilization

HCRU per patient, median (IQR) [range]	ECP (n = 20)	Non‐ECP (n = 20)	*P* value
**ECP sessions, days**	18.0 (13.0) [7–29]	0.0 (0.0) [0]	‐
**Outpatient day hospital visits, n**	3.0 (8.0) [0–19]	1.0 (2.5) [0–21]	.053
**External consultation visits, n**	15.0 (14.5) [0–20]	7.0 (10.5) [0–67]	.011
**Hospital stays, n**	0.5 (2.0) [0–7]	1.0 (1.5) [0–5]	.507
**Length of stay, days**	4.0 (42.5) [0–246]	20.5 (71.5) [0–135]	.206
**Proportion of hospitalization days, %**	1.1 (21.4) [0–67]	5.6 (13.4) [0–43]	.206
**Exposure period, days**	365 (0.0) [312–365]	365 (0.0) [21–365]	‐
**Platelet transfusion, n**	0 (4.5) [0–70]	2.0 (19.0) [0–79]	.525
**Red blood cell units, n**	0 (5.0) [0–74]	2.0 (13.0) [0–64]	.204

Abbreviations: ECP, extracorporeal photopheresis; HCRU, healthcare resource utilization; IQR, interquartile range.

When considering the use of ECP technologies, the total combined costs (direct and indirect) of using integrated vs multi‐step systems were €1110.93 and €1024.94 per session, respectively (full costing breakdown presented in Supplemental S2). Procedure kit required for integrated ECP was more expensive compared with multi‐step kits (€850.00 vs €500.00); however, multi‐step systems require higher structural, installation, regulatory, and human resources requirements compared with integrated technologies. The average session using multi‐step systems was estimated to take twice as much time compared with integrated technologies (Supplemental S3); estimated total time taken per session was 5 hours 40 minutes with the multi‐step system vs 2 hours 50 minutes using the integrated system.

### 3.3 Response and survival

Response to treatment was similar across the two cohorts (Table 3); complete and partial response was achieved by six (30%) and four (20%) patients in the ECP cohort, and by five (25%) and three (15%) in the non‐ECP cohort (*P* = .65). Disease state stabilized in six (30%) ECP‐treated patients, vs four (20%) non‐ECP‐treated patients, whilst cGvHD disease progression was observed in four (20%) ECP‐treated patients and seven (35%) non‐ECP‐treated patients. During the study period, a total of five (25%) patients died in the ECP cohort due to underlying disease relapse (n = 2), cGvHD (n = 2), and infection (n = 1); whereas eight (40%) patients died in the non‐ECP cohort due to cGvHD (n = 3) and infection (n = 5).

**TABLE 3:** Patient response and survival outcomes

	ECP (n = 20)	Non‐ECP (n = 20)	*P* value
**Overall response, n (%)**			.65
Complete response	6 (30)	5 (25)	
Partial response	4 (20)	3 (15)	
Stable disease	6 (30)	4 (20)	
Progression	4 (20)	7 (35)	
Response not evaluable due to death	0 (0)	1 (5)	
**Overall survival (%)**	15 (75)	12 (60)	.31
**Further treatment lines, n (%)**			.84
None	14 (70)	12 (60)	
1 treatment	3 (15)	3 (15)	
2 treatments	2 (10)	4 (20)	
≥3 treatments	1 (5)	1 (5)	
**CMV reactivation, n (%)**			.94
None	12 (60)	13 (65)	
Reactivation	7 (35)	6 (30)	
Infection	1 (5)	1 (5)	
**Invasive fungal disease, no, n (%)**	18 (90)	16 (80)	.38
**Cause of death during study period, n (%)**			.31
Relapse	2 (10)	0 (0)	
cGvHD	2 (10)	3 (15)	
Infection with or without cGvHD	1 (5)	5 (25)	

Abbreviations: cGvHD, graft‐vs‐host disease; CMV, cytomegalovirus; ECP, extracorporeal photopheresis.

## 4 DISCUSSION

Steroid‐refractory or steroid‐dependent cGvHD is associated with high hospitalization rates and high economic burden, as shown in this study where total treatment costs averaged €25 319 (95% CI: €17 049–€33 590) per patient from diagnosis until 1 year's treatment or relapse. Although more outpatient external consultations and day hospital visits were required, ECP treatment was associated with slightly lower overall HCRU in cGvHD, with a trend toward fewer inpatient hospitalizations and shorter LOS due to fewer major complications compared to current standard of care. These observations suggest that ECP treatment in steroid‐refractory or steroid‐dependent cGvHD might yield some resource savings to hospitals in Spain. This is aligned with previous findings from a microsimulation study, where ECP was determined to be less expensive and more clinically effective than imatinib in treating cGvHD, and more cost‐effective than rituximab using accepted Spanish willingness‐to‐pay thresholds.^10^

Beyond total costs, it is also important to consider the benefits of moving the management of care to an outpatient setting; reducing the need for inpatient care is highly desirable as it alleviates the burden on hospitals and increases efficiency, with higher patient throughput and in‐hospital resource availability.^16^, ^17^ Further, decreased inpatient care is likely to reduce the incidence of hospital‐acquired conditions, including infections, which can be particularly harmful in immunocompromised patients for whom prognosis is likely to be poor.^18^ Although there was no significant difference in overall survival between the two cohorts, notably, a greater proportion of deaths were attributable to infectious complications in the non‐ECP cohort compared with the ECP cohort. This finding could be reflective of the steroid‐sparing effect of ECP treatment compared with other second‐line treatments, and is consistent with previous studies^6^, ^19^, ^20^, ^21^; reduction in corticosteroid use is associated with an improved safety profile in short‐ and long‐term cGvHD management.^14^, ^22^ Further, due to a potentially increased number of hospitalizations and LOS, patients not receiving ECP treatment might have more exposure to nosocomial pathogens.^10^

A key strength of this study was the matched‐cohort approach that allowed for direct comparison of cost outcomes attributable to ECP vs standard therapies; however, it is noted that changes in the management of care during the study period presented difficulty in patient matching. Consequently, despite the large number of cases reviewed, relatively few patients were ultimately included in each cohort, and differences in resource utilization cannot therefore be easily upscaled. However, this was an exploratory analysis focusing on determining cost differences attributable to ECP vs standard therapies; as such, this study was not designed to be statistically powered. Additionally, given that the DRG system used to calculate hospital reimbursement costs is used nationally in Spain, our findings are likely to be applicable to other institutions across the country.

Overall, this cohort‐matched study highlights that ECP treatment may yield economic savings in Spain, potentially through both resource savings and a shift of costs toward outpatient care. The benefits of ECP might also extend to patients with fewer inpatient hospitalizations and shortened LOS owing to a potentially reduced risk of major cGvHD‐related complications. As such, ECP may help to improve quality of life for patients with cGvHD and alleviate the burden on hospitals and caregivers.^23^, ^24^ Studies using larger patient populations are required to further investigate the pharmacoeconomic impact of ECP treatment in patients with steroid‐refractory cGvHD in Spain and other geographies. In a future study, it may also be of interest to investigate the cost benefit of ECP for different subgroups of patients based on their treatment response and disease activity.

## CONFLICT OF INTEREST

B. B., A. S.‐A., O. B.‐L., J. E. M.‐V., R. R.‐V, D. M.‐C., I. G., P. S., I. L., J. L. P., J. S., I. C., E. A., A. D.‐G., J. M., A. B., N. C., G. S., M. Á. S., and P. M. have no conflicts of interest to declare.

## AUTHOR CONTRIBUTIONS

Substantial contributions to study conception and design: Blanca Boluda, Antonio Solana‐Altabella, Isafer Cano, Evelyn Acuña‐Cruz, Rebeca Rodríguez‐Veiga, Octavio Ballesta‐López, Juan Eduardo Megías‐Vericat, David Martínez‐Cuadrón, Ines Gómez, Pila Solves, Indignacio Lorenzo, Jose Luis Piñana, Jaime Sanz, Manuel Guerreiro, Juan Montoro Gómez, Alvaro Díaz‐González, Javier Marco, Ana Blanco, Miguel Á. Sanz, Pau Montesinos; substantial contributions to analysis and interpretation of the data: Blanca Boluda, Antonio Solana‐Altabella, Isafer Cano, Evelyn Acuña‐Cruz, Rebeca Rodríguez‐Veiga, Octavio Ballesta‐López, Juan Eduardo Megías‐Vericat, David Martínez‐Cuadrón, Ines Gómez, Pila Solves, Indignacio Lorenzo, Jose Luis Piñana, Jaime Sanz, Manuel Guerreiro, Juan Montoro Gómez, Alvaro Díaz‐González, Javier Marco, Ana Blanco, Miguel Á. Sanz, Pau Montesinos; drafting the article or revising it critically for important intellectual content: Blanca Boluda, Antonio Solana‐Altabella, Isafer Cano, Evelyn Acuña‐Cruz, Rebeca Rodríguez‐Veiga, Octavio Ballesta‐López, Juan Eduardo Megías‐Vericat, David Martínez‐Cuadrón, Ines Gómez, Pila Solves, Indignacio Lorenzo, Jose Luis Piñana, Jaime Sanz, Manuel Guerreiro, Juan Montoro Gómez, Alvaro Díaz‐González, Javier Marco, Ana Blanco, Miguel Á. Sanz, Pau Montesinos; final approval of the version of the article to be published: Blanca Boluda, Antonio Solana‐Altabella, Isafer Cano, Evelyn Acuña‐Cruz, Rebeca Rodríguez‐Veiga, Octavio Ballesta‐López, Juan Eduardo Megías‐Vericat, David Martínez‐Cuadrón, Ines Gómez, Pila Solves, Indignacio Lorenzo, Jose Luis Piñana, Jaime Sanz, Manuel Guerreiro, Juan Montoro Gómez, Alvaro Díaz‐González, Javier Marco, Ana Blanco, Miguel Á. Sanz, Pau Montesinos.

## Supporting information

**Appendix** S1: Supporting information

## Data Availability

Data not available.

## Endnote

* DRG 579 refers to overnight stay included, or was due to, a major complication (ie, sepsis, pneumonia, parenteral nutrition, or respiratory failure)

## References

[1] Socié G, Stone JV, Wingard JR, et al. Long‐term survival and late deaths after allogeneic bone marrow transplantation. Late effects working committee of the international bone marrow transplant registry. N Engl J Med. 1999;341:14‐21. 10387937 10.1056/NEJM199907013410103

[2] Martin PJ, Counts GW Jr, Appelbaum FR, et al. Life expectancy in patients surviving more than 5 years after hematopoietic cell transplantation. J Clin Oncol. 2010;28:1011‐1016. 20065176 10.1200/JCO.2009.25.6693 PMC2834427

[3] Socié G, Ritz J. Current issues in chronic graft‐versus‐host disease. Blood. 2014;124:374‐384. 24914139 10.1182/blood-2014-01-514752 PMC4102710

[4] Wingard JR, Majhail NS, Brazauskas R, et al. Long‐term survival and late deaths after allogeneic hematopoietic cell transplantation. J Clin Oncol. 2011;29:2230‐2239. 21464398 10.1200/JCO.2010.33.7212 PMC3107742

[5] Penack O, Marchetti M, Ruutu T, et al. Prophylaxis and management of graft versus host disease after stem‐cell transplantation for haematological malignancies: updated consensus recommendations of the European society for blood and marrow transplantation. Lancet Haematol. 2020;7:e157‐e167. 32004485 10.1016/S2352-3026(19)30256-X

[6] Wolff D, Gerbitz A, Ayuk F, et al. Consensus conference on clinical practice in chronic graft‐versus‐host disease (GVHD): first‐line and topical treatment of chronic GVHD. Biol Blood Marrow Transplant. 2010;16:1611‐1628. 20601036 10.1016/j.bbmt.2010.06.015

[7] Ferrara JLM, Levine JE, Reddy P, Holler E. Graft‐versus‐host disease. Lancet. 2009;373:1550‐1561. 19282026 10.1016/S0140-6736(09)60237-3 PMC2735047

[8] Buchman AL. Side effects of corticosteroid therapy. J Clin Gastroenterol. 2001;33:289‐294. 11588541 10.1097/00004836-200110000-00006

[9] Hart JW, Shiue LH, Shpall EJ, Alousi AM. Extracorporeal photopheresis in the treatment of graft‐versus‐host disease: evidence and opinion. Ther Adv Hematol. 2013;4:320‐334. 24082993 10.1177/2040620713490316 PMC3766348

[10] Abu‐Dalle I, Reljic T, Nishihori T, et al. Extracorporeal photopheresis in steroid‐refractory acute or chronic graft‐versus‐host disease: results of a systematic review of prospective studies. Biol Blood Marrow Transplant. 2014;20:1677‐1686. 24867779 10.1016/j.bbmt.2014.05.017

[11] Sakellari I, Gavriilaki E, Batsis I, et al. Favorable impact of extracorporeal photopheresis in acute and chronic graft versus host disease: prospective single‐center study. J Clin Apher. 2018;33:654‐660. 30394564 10.1002/jca.21660

[12] Gandelman JS, Song DJ, Chen H, et al. A prospective trial of extracorporeal photopheresis for chronic graft‐versus‐host disease reveals significant disease response and no association with frequency of regulatory T cells. Biol Blood Marrow Transplant. 2018;24:2373‐2380. 29981848 10.1016/j.bbmt.2018.06.035

[13] de Waure C, Capri S, Veneziano MA, et al. Extracorporeal photopheresis for second‐line treatment of chronic graft‐versus‐host diseases: results from a health technology assessment in Italy. Value Health. 2015;18:457‐466. 26091600 10.1016/j.jval.2015.01.009

[14] Drexler B, Buser A, Infanti L, Stehle G, Halter J, Holbro A. Extracorporeal photopheresis in graft‐versus‐host disease. Transfus Med Hemother. 2020;47:214‐224. 32595426 10.1159/000508169 PMC7315199

[15] Clopper CJ, Pearson ES. The use of confidence or fiducial limits illustrated in the case of the binomial. Biometrika. 1934;26:404‐413.

[16] Carey K, Stefos T. Measuring inpatient and outpatient costs: a cost‐function approach. Health Care Financ Rev. 1992;14:115‐124. 10127447 PMC4193307

[17] Grannemann TW, Brown RS, Pauly MV. Estimating hospital costs: a multiple‐output analysis. J Health Econ. 1986;5:107‐127. 10287222 10.1016/0167-6296(86)90001-9

[18] Mendes ET, Dulley F, Basso M, et al. Healthcare‐associated infection in hematopoietic stem cell transplantation patients: risk factors and impact on outcome. Int J Infect Dis. 2012;16:e424‐e428. 22463920 10.1016/j.ijid.2012.01.015

[19] Pierelli L, Perseghin P, Marchetti M, et al. Extracorporeal photopheresis for the treatment of acute and chronic graft‐versus‐host disease in adults and children: best practice recommendations from an Italian society of hemapheresis and cell manipulation (SIdEM) and Italian group for bone marrow transplantation (GITMO) consensus process. Transfusion. 2013;53:2340‐2352. 23305044 10.1111/trf.12059

[20] Ussowicz M, Musiał J, Mielcarek M, et al. Steroid‐sparing effect of extracorporeal photopheresis in the therapy of graft‐versus‐host disease after allogeneic hematopoietic stem cell transplantation. Transplant Proc. 2013;45:3375‐3380. 24182819 10.1016/j.transproceed.2013.07.053

[21] Greinix HT, Worel N, Just U, Knobler R. Extracorporeal photopheresis in acute and chronic graft‐versus‐host disease. Transfus Apher Sci. 2014;50:349‐357. 24780392 10.1016/j.transci.2014.04.005

[22] Waljee AK, Rogers MAM, Lin P, et al. Short‐term use of oral corticosteroids and related harms among adults in the United States: population based cohort study. BMJ. 2017;357:j1415. 28404617 10.1136/bmj.j1415 PMC6284230

[23] Flowers MED, Apperley JF, van Besien K, et al. A multicenter prospective phase 2 randomized study of extracorporeal photopheresis for treatment of chronic graft‐versus‐host disease. Blood. 2008;112:2667‐2674. 18621929 10.1182/blood-2008-03-141481

[24] Greinix HT, Socié G, Bacigalupo A, et al. Assessing the potential role of photopheresis in hematopoietic stem cell transplant. Bone Marrow Transplant. 2006;38:265‐273. 16883310 10.1038/sj.bmt.1705440

