# Supplementary material for: Extracorporeal photopheresis vs standard therapies for steroid‐refractory chronic graft‐vs‐host disease: Pharmacoeconomic assessment of hospital resource use in Spain
Source: J Clin Apher. 2021 May 8;36(4):612–20. doi: 10.1002/jca.21901 (PMC8453768; doi:10.1002/jca.21901)
Supplement: Supplementary file 1 — Appendix S1: Supporting information [file JCA-36-612-s001.docx]

**Extracorporeal photopheresis vs standard therapies for steroid-refractory chronic graft-versus-host disease: pharmacoeconomic assessment of hospital resource use in Spain**

**Short title:** Pharmacoeconomic Assessment of ECP in Spain

**Key words:** Extracorporeal photopheresis; healthcare resource utilisation; cost comparison; graft-versus-host disease.

**SUPPLEMENTAL DATA**

Supplemental S1. Resource costs

| **Resource** | **Cost (€)** |
| --- | --- |
| External consultation^a^ | 108 |
| Outpatient day’s stay at a day hospital^a^ | 691 |
| Overnight stay included, or was due to, a major complication (i.e. sepsis, pneumonia, parenteral nutrition or respiratory failure) (DRG 579) | 22,383 |
| Overnight stay was not associated with a major complication in haematologic patients (DRG 875) | 5,154 |

^a^Costs of external consultations and day’s stay at a day hospital were sourced from Oblikue Esalud Database, available at: esalud.oblikue.com [accessed 11.09.2020]. DRG: diagnosis-related group.

Supplemental S2. Cost of multi-step versus integrated ECP technologies

|  | Cost (€) | |
| --- | --- | --- |
| ECP-associated expenditure | Integrated system | Multi-step system |
| Physician base salary (per session) | 6.16 | 6.16 |
| Technical operator base salary (per session) | 25.80 | 55.04 |
| Bed retention | 36.63 | 73.26 |
| Procedure kit I | 850.00 | 250.00 |
| Procedure kit II | - | 250.00 |
| Laboratory analysis (patient sample) | 86.30 | 86.30 |
| Methoxalen (Uvadex® vs S.A.L.F. 8-MOP) | 75.00 | 45.00 |
| Cell count after cell collection (haematocrit and leukocyte population) | - | 20.46 |
| Biological analysis after irradiation | - | 52.48 |
| Indirect cost (labelling, material transportation, sampling, equipment maintenance) | 31.04 | 186.24 |
| Total cost per session | 1,110.93 | 1,024.94 |

8-MOP: 8-methoxypsoralen; ECP: extracorporeal photopheresis.

**Supplemental S3.** Estimated time taken for each stage of ECP with multi-step versus integrated ECP technologies

|  | Time associated with ECP (min) | |
| --- | --- | --- |
| ECP stage | Integrated system | Multi-step system |
| Set-up | 30 | 30 |
| Apheresis | 60 | 100 |
| Illumination | 50 | 160 |
| Reinfusion | 10 | 30 |
| Additional bed retention | 20 | 20 |
| *Physician* | *20* | *20* |
| *Technical operator* | *150* | *320* |
| Total time per session | 170 | 340 |

Technical operator time is the sum of time taken for set-up, apheresis, illumination and reinfusion. Physician time is spent during additional bed retention. ECP: extracorporeal photopheresis.
